# Does age moderate the influence of early life language experiences? A Naturalistic home observation study

**DOI:** 10.1016/j.ecresq.2023.01.009

**Published:** 2023-03-02

**Authors:** Katrina d'Apice, Sophie von Stumm

**Affiliations:** a Population Health Sciences, Bristol Medical School, University of Bristol, Canynge Hall, 39 Whatley Road, Bristol BS8 2PS, United Kingdom; b Department of Education, University of York, United Kingdom

**Keywords:** Home observation, Language, Cognitive development, Parent-child Interaction

## Abstract

We explored if children's age moderated associations between their early life language experiences and their linguistic and cognitive skills. For 107 British children, aged 24 to 48 months, and their families, we collected 3 day-long audio-recordings of their naturalistic home environments (*M* = 15.06 h per day, *SD* = 1.87). Children's cognitive ability was assessed by parent-ratings and with a cognitive testing booklet that children completed at home. We found that the quantity, lexical diversity and vocabulary sophistication of adult speech were associated with children's linguistic and cognitive skills. However, these associations were not moderated by children's age. Our findings suggest that the influence of early life language experience is not differentiated at age 24 to 48 months, at least in the current sample.

Children's exposure to adult speech in early life is pivotal for their own language and cognitive development (Caskey et al., 2014; Hart & Risley, 1995; Hoff, 2003). In particular, previous studies identified three characteristics of adult speech, including (i) the quantity (d'Apice et al., 2019; Hart & Risley, 1995; Weisleder & Fernald, 2013), (ii) the lexical diversity (Pan et al., 2005), and (iii) the vocabulary sophistication (Rowe, 2012), that inform children's language and cognitive development. The influence of these characteristics of adult speech on children's language and cognitive outcomes has been suggested to vary as a function of children's age (Jones & Rowland, 2017; Rowe, 2012), but corresponding findings are scarce and inconsistent. The current study uses extensive naturalistic home observations to add to the existing body of research on interactions between experience and development, overcoming some of the limitations of previous research in this area.

Children who are exposed to a large quantity of adult speech in the early stages of language acquisition experience multiple repetitions of high-frequency words. These repetitions result in a reduction of processing time for encoding, which aids vocabulary development (Ellis, 2002). After children have built up a principal vocabulary base, exposure to adult speech that is rich in diversity and sophistication helps to further enhance children's lexicons by introducing them to new and rare words (Jones & Rowland, 2017; Rowe, 2012). Therefore, the quantity of adult speech is likely to influence children's early language acquisition, but as their linguistic competence develops, the lexical diversity and vocabulary sophistication of adult speech becomes more relevant for children's language development. In this case, we would expect for age to moderate the influence of the characteristics of adult speech on children's language development.

Constructivist theories of language acquisition suggest that learning is a gradual process whereby children construct new knowledge by incorporating into their current knowledge base new information they are exposed to (Behrens, 2021). Therefore, it is important to understand which aspects of children's language environments are most influential at different child ages to ensure children develop to their full potential.

We have identified three previous studies that explored whether children's age moderates the association between characteristics of adult speech and children's language. First, Pan and colleagues (2005) videotaped 10 min interactions of 108 U.S. mother-child dyads in the family home when the child was aged 14, 24 and 36 months. At each observation, mothers could choose from books and age-appropriate toys to use for engaging with their child; the quantity and the lexical diversity of mothers’ speech (i.e., word types) during these interactions was assessed. In a series of multi-level models, the lexical diversity of mothers’ speech was significantly associated with children's growth in lexical diversity over time. Furthermore, there was a significant interaction between children's age and their mothers’ lexical diversity in the prediction of children's language development. Specifically, at age 14 months, children whose mothers used high levels of lexically diverse speech (i.e., in the 90th percentile) did not differ in their own lexical diversity from children whose mothers spoke with low levels of lexical diversity (i.e., in the 10th percentile). By the age of 24 months, children of high-diversity mothers produced on average 9-word types more than children of low-diversity mothers. But by age 36 months, the difference between children from high- and low-diversity mothers had diminished to 1-word type (Pan et al., 2005), suggesting that the influence of mothers’ lexical diversity on their children's lexical diversity is most pronounced around 24 months of age. By contrast to the association between mothers’ and children's lexical diversity, Pan et al. (2005) observed no effect of mothers’ quantity of words on children's linguistic growth.

In a second, later study, Rowe (2012) reported findings based on analyses of 90 min video recordings of 50 parent-child dyads undergoing normal daily activities in their homes that were collected at the children's ages of 18, 30 and 42 months. Children's receptive vocabulary was assessed 1 year after each home observation was conducted. Children's growth in receptive vocabulary at age 30 and 54 months was not predicted by either the quantity, lexical diversity, or vocabulary sophistication of parents’ speech, after controlling for confounding variables. However, at 42 months, the diversity and sophistication of parents’ words spoken a year earlier were both significantly associated with children's growth in receptive vocabulary, accounting independently for 9% and 6% of the variance, respectively. These findings are only partially aligned with the previous study's result that the association between mothers’ and children's lexical diversity is most pronounced at age 24 months.

The third study that assessed whether children's age moderated associations between adult speech and children's outcomes analyzed transcripts from a corpus of natural language data, known as CHILDES (MacWhinney, 2000). In this study, Jones and Rowland (2017) analyzed 2 h long audio-recordings that were collected from 16 mother-child dyads every 3 weeks over the course of one year, starting when the child was 22 months old. The number of words (i.e., the quantity of language input) and the number of word types (i.e., the lexical diversity) that mothers spoke was computed from the transcripts at child ages 22 and 28 months, and child lexical diversity was assessed when they were aged 28 and 33 months. The quantity of mothers’ language input at 22 and 28 months was not associated with children's lexical diversity at 28 or 33 months. Likewise, mothers’ lexical diversity at children's age of 22 and 28 months was not significantly related to children's gains in lexical diversity at age 28 months. However, five months later (i.e., at age 33 months), mothers’ lexical diversity at age 22 and 28 months accounted for 18% and 13% of the variance in children's growth in lexical diversity, respectively. Jones and Rowland (2017) also used computational modelling to hold the quantity of language input constant whilst varying the diversity of input, and then repeated the process but instead varied the quantity and kept the diversity of language input constant. They found that initially the model learnt more lexical items from input with a greater quantity, however this was superseded by a rich diversity of input in the latter stages of learning. The authors suggest that diverse input includes a larger number of phonemes or sub-lexical chunks from which the model is able to extrapolate to newly encountered words.

Overall, the three prior studies reported that adults’ lexical diversity predicted children's linguistic growth, but each study identified this association at a different child age. In addition, one study found moderation effects of children's age on the association between adults’ vocabulary sophistication and children's linguistic growth. Because the other two studies did not assess this language marker, they could not corroborate the finding.

We propose that the discrepancies in the earlier findings are likely to be due to three factors. First, two of the studies were based on small sample sizes (Jones & Rowland, 2017; Rowe, 2012), which have low power to detect differences in correlation strengths (Asendorpf et al., 2013). This problem affects many studies in language development because the demand for power conflicts with the high costs in time, effort, and funding for collecting data from children for developmental research.

A second reason for the previous studies’ differences in findings may be their analytical approaches. Rowe (2012) and Jones and Rowland (2017) tested associations between mothers’ and children's lexical diversity at different ages and then compared the strength of association across ages. By comparison, Pan et al. (2005) used individual growth modelling to test if mothers’ lexical diversity influenced a change in children's lexical diversity over time using child age as the measure of time.

Finally, the studies differed in the methods of observation for collecting data from the parent-child dyads. In two of the studies, a researcher visited the family home and operated a hand-held video-camera to document the parent-child interactions (Pan et al., 2005; Rowe, 2012). It is plausible that observer and social desirability biases influence parents linguistic behavior when they are aware of being observed (Gardner, 2000; Saini & Polak, 2014; Zegiob et al., 1975). The third study audio-recorded the mother-child dyads in the home, and the authors do not state if a researcher was physically present to conduct the recordings.

## The current study

Studies with larger sample sizes that specifically test for interactions using naturalistic observation data are key to further our understanding of factors that influence children's language development (Purpura, 2019). We report here data from a sample 107 family-child dyads, which affords adequate power and is the largest naturalistic home observational study of British families to date. We unobtrusively audio-recorded 107 families with the Language Environment Analysis (LENA) system (LENA Research Foundation, 2012). Each family completed 3 full days of audio-recordings and had at least one child aged 24 to 48 months, capturing a wide window of development. Our 3-daylong observation period increases the possibility of capturing meaningful differences in language input between children because the families’ behaviour is more natural than with short observations.

Extending previous work, we assessed multiple markers of children's cognitive and language outcomes and of their early life language experiences. In contrast to previous studies in this area that predominantly focused on the role of maternal speech, we included all adult speakers in our analysis to provide a realistic view of children's natural language experiences in the family home (Ramírez-Esparza et al., 2014). Also, by contrast to the previous studies, our sample was only assessed once in time with the participating children ranging in age from 24 to 48 months. We therefore model contemporaneous associations between the characteristics of adult speech and children's verbal and cognitive skills and test if these associations vary as a function of child age.

We hypothesized that the association between the quantity of adult speech and children's language would not be moderated by children's age because none of the previous studies found such an interaction. However, we expected to find that the relation between lexical diversity and vocabulary sophistication of adult speech with children's language outcomes would vary as a function of children's age. With regards to children's non-verbal cognitive outcomes, our analyses were exploratory.

## Method

### Sample

The sample comprised 107 monolingual English families with a typically developing child aged 24 to 48 months, including 105 mothers (mean age in years = 37.11, *SD* = 4.56, min = 24.48, max = 51.57), 73 fathers (mean age in years = 39.49, *SD* = 5.16, min = 25.24, max = 55.09), and 107 children (mean age in years = 2.77, *SD* = 0.55, min = 2.03, max = 3.99; 51 girls; full details on recruitment and attrition figures are reported in d'Apice et al. (2019). Most parents were native English speakers and had been born in Britain (99% and 86% respectively). The majority of mothers (58) were in part-time employment, 28 were full-time parents, 11 were employed full-time, 4 were on maternity leave, and 4 were students. For the fathers, 59 were in full-time employment, 10 in part-time roles and 4 were full-time parents. Most parents were married (96%) and held university degrees (86% of mothers and 76% of fathers). The sample was on average of high socioeconomic status (SES) although there was within-sample variation.

### Procedure

Parents first reported their demographic and child's characteristics via an online survey. Each family then received a hand-delivered box containing (a) 3 LENA audio-recorders, (b) 3 LENA children's clothes, and (c) a Parent Report of Children's Abilities (PARCA) booklet. Parents were instructed to independently audio-record for 3 days when they were mainly at home with their child. Parents were informed that LENA audio-recorders can record for up to 16 h and hence, they were instructed to start recording first thing in the morning and leave the recording on all day. On average families recorded for 15.06 h per day (*SD* = 1.87). Parents were also instructed to administer the PARCA booklet at home to their child at a convenient time. Each family received a child LENA t-shirt for their participation, and 79 families also received £50 cash, which only became available after the first families had participated due to changes in the study's funding. There was no difference in any of the variables between those families that did and those that did not receive monetary compensation.

### Measures

#### Language

***Adult word counts.*** LENA audio-recorders were “worn” by the children in the front pocket of custom-made clothing. These small, lightweight devices recorded all sounds within a six-foot radius of the study child for up to 16 h per day. The total number of words that a child heard from all adults in their environment over the 3 recording days were extracted using LENA pro software version V3.4.0 –143. Acceptable inter-rater reliability (Cohen's *k* 0.65) between LENA- and human-derived adult word counts has been reported in a sample of 70 12-hr recordings (Zimmerman et al., 2009).

***Lexical diversity.*** We selected two 5 min audio excerpts per day between 8am and 11 am, and 5 pm and 8 pm that registered the highest conversational turn count (CTC) in LENA (i.e., 2 × 5 min, across 3 days, totaling 30 min per family). Conversational turns refer to adult-child interactions in which one speaker initiates a conversation and the other responds within 5 s. We decided to select audio excerpts for transcription with the highest CTC within the morning and evening to capture the richest adult-child interactions. We only used automated CTC to select excerpts, however it should be noted that several studies suggest LENA CTC are less reliable than LENA adult word counts and thus caution should be taken if using automated CTC in research (Busch et al., 2018; Ramirez et al., 2021). Because LENA CTC are not based on the content of speech, it is possible that they may not reflect actual conversational turns (i.e., initiation-response pairs), just different speakers’ utterances that occur within 5 s of one another.

Overall, 92 families had 6 audio excerpts and 15 families had 4 or 5 audio excerpts available for transcription. The audio excerpts were transcribed by professional typists using the Codes for Human Analysis of Transcripts (CHAT; Macwhinney, 2000) and proofread by two trained research assistants. Each family's transcripts (i.e., six in total) were combined and all adult speakers were assigned the same code. The combined transcripts were subjected to VOCD analysis in the Computerized Language Analysis (CLAN; MacWhinney, 2000) program, which generates D scores. D scores estimate the probability of a speaker introducing a new word into successively longer samples of speech and as such represents lexical diversity. Study child D scores were computed in the same way as adult D scores, by performing VOCD analysis on the combined transcripts.

**V*ocabulary sophistication.*** From the combined transcripts, described above (i.e., 30 min per family), we removed all words on the Dale-Chall word list, as well as their inflected forms, and all non-dictionary words, including names of family members, friends and pets. The Dale-Chall word list (Chall & Dale, 1995; Dale & Chall, 1948), lists the 3,000 most common words known by fourth graders. The remaining words in the transcripts were considered sophisticated words, and hence we computed the number of sophisticated word types (i.e., number of different sophisticated words) that were uttered by the adults within a family. To control for volubility, we divided the number of adult sophisticated word types by the total number of adult word types. Likewise, children's vocabulary sophistication was calculated as the proportion of the child's sophisticated word types from their total word types. This method has been used in previous research (Rowe, 2012; Weizman & Snow, 2001).

**Children's cognitive ability.** The PARCA was used to measure children's non-verbal cognitive ability such as shape awareness, reasoning, and problem solving (Oliver et al., 2002; Saudino et al., 1998). The PARCA comprises two components: a parent report and a parent administered testing booklet. For the online parent report, parents responded to 28 questions such as “Can your child stack seven small blocks on top of each other by him or herself?” with either yes, no, or I don't know. Parent report ratings showed good reliability (Cronbach's alpha = .72) and were therefore summed.

The PARCA booklet was administered at home by the parent, and included 9 drawing, 7 copying and 10 matching tasks. Responses were assessed independently by 2 trained research assistants according to the test scoring guidelines (Oliver et al., 2002; Saudino et al., 1998). Initial inter-rater agreement of 92.9% rose to 100% after discussion with reference to the scoring instructions. Composite scores from the drawing, copying and matching tasks, which correlated .33, .42, and .51, were *z* transformed and summed. The 3 tasks showed good internal consistency (Cronbach's alpha = .68).

The total PARCA has been validated against the Mental Development Index of the Bayley Scales of Infant Development-II (Bayley, 1993) and the nonverbal component of the McCarthy Scales of Children's Abilities (McCarthy, 1972) in a sample of 85 3-year-olds (*r* = .54, *p* < .001, and *r* = .51, *p* < .001 respectively; Oliver et al., 2002). A revised version of the PARCA is part of the National Institute for Health and Care Excellence's (2017) guidelines for assessing child development, which supports the notion that parents can accurately report on their children's abilities (Blaggan et al., 2014; Martin et al., 2013).

**Socioeconomic status.** SES was indexed by three markers: (1) Education: parents reported the highest educational qualification they had obtained (school leaving certificate, national vocational qualification, undergraduate degree, or postgraduate degree). (2) McArthur Scale of Subjective Social Status (Adler et al., 2000): parents were shown a ladder with 10 rungs and the following text “Think of this ladder as representing where people stand in our society. At the top of the ladder are the people who are the best off, those who have the most money, most education, and best jobs. At the bottom are the people who are the worst off, those who have the least money, least education, and worst jobs or no job.” The parents were asked to indicate which rung of the ladder best represented their own status in society. (3) Overcrowding score: the number of people living in the family home was divided by the number of rooms in the household, so that a higher value indicated less overcrowding. The three SES markers were *z* transformed and summed. Where data was available for both parents in a family, responses were averaged.

### Statistical analysis

Only recording hours that contained at least one adult word were included in our analyses to exclude times when the child was sleeping. Recording duration was regressed onto adult word counts, and standardized residuals were saved and used in the subsequent analyses. The number of excerpts transcribed (range 4 to 6 across families) was regressed onto both the adults’ and children's lexical diversity and vocabulary sophistication measures, and standardized residuals were saved.

To evaluate if children's age moderated the association between the adult language markers and children's cognitive and language outcomes, we generated a series of stepwise regression models that included the relevant interaction terms (i.e., children's age x early life language experience). We fitted an independent model for each child outcome measure (i.e., lexical diversity, vocabulary sophistication, PARCA booklet and PARCA ratings). The predictors were added to each model in the following order: First, we included child gender and family SES. Next, we added children's age, followed by the three characteristics of adult speech (i.e., adults’ word counts, lexical diversity, and their vocabulary sophistication). We tested each interaction term in a separate model, including children's age*adults’ word counts, children's age*adults’ lexical diversity, and children's age*adults’ vocabulary sophistication. To adjust for multiple comparisons, the *p* value for each model was divided by 12 (i.e., 4 child outcomes x 3 interaction terms). Likewise, we report the 99.6 % confidence interval for all predictors, equivalent to *p* = .004.

## Results

Descriptive statistics for all study variables are displayed in Table 1. Children's age significantly correlated with children's lexical diversity, the PARCA book and the parent report but not with the adult language markers (range = .43 to .66; Table 2). Adults’ lexical diversity correlated .40 with vocabulary sophistication, supporting the measures’ concurrent validity. However, adults’ word counts were associated neither with adults’ lexical diversity nor with their vocabulary sophistication, because adults’ word counts reflect input from a larger number of speakers than the other adult language measures. Children's vocabulary sophistication correlated .14 with their lexical diversity.

**Table 1:** Descriptive statistics of all study variables.

Variable	*M*	*SD*	Minimum	Maximum
SES index	-.02	.56	-1.69	.95
Children's age in years^a^	2.74	.55	2.03	3.99
Adults’ word counts^b^	18,021.70	7148.62	2953.65	44,652.22
Adults’ lexical diversity^c^	34.26	8.41	10.54	54.72
Adults’ vocabulary sophistication^c^	0	.03	-.08	.13
Children's word counts^e^	611.95	271.55	58	1431
Children's lexical diversity^c^	21.31	13.37	-12.49	54.42
Children's vocabulary sophistication^c^	.05	.04	-.02	.24
PARCA book – standardized	0	.78	-1.82	1.62
Parent report	19.41	3.31	12	27

*Note.* Descriptives are based on complete data *N* = 107 except where indicated otherwise. PARCA = Parent Report of Children's Abilities; SES = socioeconomic status

a average across recording days. Variables corrected for ^b^ recording duration, and ^c^ number of available recordings. ^d^*N* = 104. ^e^ From the combined transcripts (i.e., 30 min per child).

**Table 2:** Pairwise correlations between all study variables.

	1	2	3	4	5	6	7	8	9
1. Child gender	-								
2. Children's age	-.17	-							
3. SES index	.02	-.06	-						
4. Adults’ word counts^a^	-.13	.06	.11	-					
5. Adults’ lexical diversity^b^	.06	-.11	.07	-.07	-				
6. Adults’ vocabulary sophistication^b^	.13	.08	.15	.14	**.40**	-			
7. Children's lexical diversity^b^	**-.21**	**.43**	.17	.17	.19	.14	-		
8. Children's vocabulary sophistication^b^	.08	.12	.04	.06	.09	**.49**	.15	-	
9. PARCA book^c^	**-.20**	**.66**	.08	**.35**	.07	**.21**	**.48**	**.25**	-
10. Parent report	**-.26**	**.55**	.07	.13	-.09	.14	**.39**	.08	**.49**

*Note.* Based on complete data *N* = 107 except where indicated otherwise. PARCA = Parent Report of Children's Abilities; SES = socioeconomic status. Variables corrected for ^a^ recording duration

b number of available recordings. ^c^*N* = 104. Correlations significant at *p* < .05 are shown in bold.

Stepwise regression analyses predicting children's lexical diversity are displayed in Tables 3 and 4. Children's age consistently predicted children's lexical diversity, accounting for 18% to 20% of the variance across the models. However, no significant interactions between children's age and the adult language markers were observed. Tables 5 and 6 show the regression models for children's vocabulary sophistication. Adults’ vocabulary sophistication predicted children's vocabulary sophistication, accounting for 28% of the variance, but like before with regard to lexical diversity, there was no interaction with children's age. For children's cognitive outcomes, Tables 7 and 8 display the regression models for the PARCA book. Both children's age and adults’ word counts consistently predicted PARCA book scores, accounting for 41% and 8% of the variance respectively. However, children's age did not moderate the association between adults’ word counts and PARCA book scores. For the parent report, children's age explained between 24% to 27% of the variance across the models, yet no other associations were found (Tables 9 and 10). Significant interaction terms were neither observed at the conventional alpha level of *p* < .05 nor at the level of *p* < .004 that was adjusted for multiple comparison.

**Table 3:** Stepwise regression models predicting children's lexical diversity.

	Children's Lexical Diversity											
	Model 1				Model 2				Model 3			
	*B*	*SE* B	β	99.6% CI	*B*	*SE* B	β	99.6% CI	*B*	*SE* B	β	99.6% CI
Gender	-.43	.19	-.22	[-1.00, .12]	-.29	.17	-.15	[-.81, .22]	-.27	.17	-.14	[-.78, .23]
SES	.31	.17	.17	[-.19, .80]	.35	.15	.20	[-.10, .80]	.30	.15	.17	[-.14, .75]
Children's age					**.42**	**.09**	**.42**	**[.16, .68]**	**.44**	**.09**	**.44**	**[.19, .69]**
AWC^a^									.05	.04	.13	[-.05, .16]
ALD^b^									.25	.09	.25	[-.02, .52]
AVS^b^									-.03	.09	-.03	[-.31, .25]
Age*AWC^a^												
Age*ALD^b^												
Age*AVS^b^												
*R*^2^	.06			.22			.27					
*F*	4.23			11.04			7.53					
*p*	0.20			< .001			< .001					

*Note.* SES = socioeconomic status; AWC = adults’ word counts; ALD = adults’ lexical diversity; AVS = adults’ vocabulary sophistication; CI = confidence interval. Variables corrected for ^a^ recording duration

b number of available recordings. Predictors significant at *p* < .004 are shown in bold. Each models’ *p* value is adjusted for 12 comparisons.

**Table 4:** Stepwise regression models predicting children's lexical diversity.

	Children's Lexical Diversity											
	Model 4				Model 5				Model 6			
	*B*	*SE* B	β	99.6% CI	*B*	*SE* B	β	99.6% CI	*B*	*SE* B	β	99.6% CI
Gender	-.28	.17	-.14	[-.79, .23]	-.28	.17	-.14	[-.79, .23]	-.28	.17	-.14	[-.79, .23]
SES	.28	.15	.16	[-.17, .72]	.31	.15	.17	[-.14, .75]	.30	.15	.17	[-.14, .75]
Children's age	**.43**	**.09**	**.43**	**[.17, .68]**	**.45**	**.09**	**.45**	**[.19, .71]**	**.44**	**.09**	**.44**	**[.18, .69]**
AWC^a^	.05	.04	.12	[-.05, .15]	.05	.04	.13	[-.05, .16]	.05	.04	.13	[-.05, .16]
ALD^b^	.25	.09	.25	[-.02, .52]	.26	.09	.26	[-.02, .53]	.25	.09	.25	[-.02, .52]
AVS^b^	-.02	.09	-.02	[-.30, .26]	-.03	.09	-.03	[-.31, .25]	-.03	.10	-.03	[-.31, .25]
Age*AWC^a^	.04	.03	.11	[-.05, .14]								
Age*ALD^b^					.05	.09	.05	[-.20, .31]				
Age*AVS^b^									.03	.07	.04	[-.17, .24]
*R*^2^	.28			.27			.26					
*F*	6.74			6.47			6.44					
*p*	< .001			< .001			< .001					

*Note.* SES = socioeconomic status; AWC = adults’ word counts; ALD = adults’ lexical diversity; AVS = adults’ vocabulary sophistication; CI = confidence interval. Variables corrected for ^a^ recording duration

b number of available recordings. Predictors significant at *p* < .004 are shown in bold. Each models’ *p* value is adjusted for 12 comparisons.

**Table 5:** Stepwise regression models predicting children's vocabulary sophistication.

	Children's Vocabulary Sophistication											
	Model 1				Model 2				Model 3			
	*B*	*SE* B	β	99.6% CI	*B*	*SE* B	β	99.6% CI	*B*	*SE* B	β	99.6% CI
Gender	.16	.20	.08	[-.42, .73]	.21	.20	.10	[-.38, .79]	.06	.18	.03	[-.47, .59]
SES	.06	.17	.04	[-.45, .58]	.08	.17	.04	[-.43, .59]	-.05	.16	-.03	[-.52, .41]
Children's age					.14	.10	.14	[-.15, .44]	.07	.09	.07	[-.19, .34]
AWC^a^									-.01	.04	-.01	[-.11, .10]
ALD^b^									-.11	.10	-.11	[-.39, .17]
AVS^b^									**.53**	**.10**	**.53**	**[.24, .82]**
Age*AWC^a^												
Age*ALD^b^												
Age*AVS^b^												
*R*^2^	-0.01			0			.21					
*F*	0.40			0.97			5.71					
*p*	8.08			4.89			< .001					

*Note.* SES = socioeconomic status; AWC = adults’ word counts; ALD = adults’ lexical diversity; AVS = adults’ vocabulary sophistication; CI = confidence interval. Variables corrected for ^a^ recording duration

b number of available recordings. Predictors significant at *p* < .004 are shown in bold. Each models’ *p* value is adjusted for 12 comparisons.

**Table 6:** Stepwise regression models predicting children's vocabulary sophistication.

	Children's Vocabulary Sophistication											
	Model 4				Model 5				Model 6			
	*B*	*SE* B	β	99.6% CI	*B*	*SE* B	β	99.6% CI	*B*	*SE* B	β	99.6% CI
Gender	.06	.18	.03	[-.47, .59]	.07	.18	.04	[-.46, .60]	.06	.18	.03	[-.47, .59]
SES	-.06	.16	-.03	[-.53, .41]	-.06	.16	-.03	[-.52, .40]	-.05	.16	-.03	[-.52, .41]
Children's age	.07	.09	.07	[-.20, .34]	.05	.09	.05	[-.22, .33]	.07	.09	.07	[-.19, .34]
AWC^a^	-.01	.04	-.01	[-.12, .10]	-.01	.04	-.02	[-.12, .10]	-.01	.04	-.01	[-.12, .10]
ALD^b^	-.11	.10	-.11	[-.39, .17]	-.12	.10	-.12	[-.41, .16]	-.11	.10	-.11	[-.40, .17]
AVS^b^	**.53**	**.10**	**.53**	**[.24, .82]**	**.53**	**.10**	**.53**	**[.24, .82]**	**.53**	**.10**	**.53**	**[.23, .82]**
Age*AWC^a^	.01	.03	.02	[-.09, .11]								
Age*ALD^b^					-.08	.09	-.08	[-.35, .18]				
Age*AVS^b^									.01	.07	.01	[-.21, .22]
*R*^2^	.20			.21			.20					
*F*	4.86			5.02			4.85					
*p*	< .01			< .001			< .01					

*Note.* SES = socioeconomic status; AWC = adults’ word counts; ALD = adults’ lexical diversity; AVS = adults’ vocabulary sophistication; CI = confidence interval. Variables corrected for ^a^ recording duration

b number of available recordings. Predictors significant at *p* < .004 are shown in bold. Each models’ *p* value is adjusted for 12 comparisons.

**Table 7:** Stepwise regression models predicting PARCA book.

	PARCA Book^c^											
	Model 1				Model 2				Model 3			
	*B*	*SE* B	β	99.6% CI	*B*	*SE* B	β	99.6% CI	*B*	*SE* B	β	99.6% CI
Gender	-.32	.15	-.20	[-.76, .13]	-.16	.12	-.10	[-.51, .18]	-.14	.11	-.09	[-.46, .17]
SES	.13	.14	.09	[-.28, .54]	.10	.11	.07	[-.21, .42]	.04	.10	.03	[-.25, .32]
Children's age					**.53**	**.06**	**.64**	**[.35, .71]**	**.52**	**.06**	**.64**	**[.36, .69]**
AWC^a^									**.09**	**.02**	**.29**	**[.03, .16]**
ALD^b^									.10	.06	.13	[-.07, .27]
AVS^b^									.07	.06	.09	[-.11, .25]
Age*AWC^a^												
Age*ALD^b^												
Age*AVS^b^												
*R*^2^	.03			.43			.55					
*F*	2.56			27.41			21.60					
*p*	1.00			< .001			< .001					

*Note.* PARCA = Parent Report of Children's Abilities; SES = socioeconomic status; AWC = adults’ word counts; ALD = adults’ lexical diversity; AVS = adults’ vocabulary sophistication; CI = confidence interval. Variables corrected for ^a^ recording duration

b number of available recordings. ^c^*N* = 104. Predictors significant at *p* < .004 are shown in bold. Each models’ *p* value is adjusted for 12 comparisons.

**Table 8:** Stepwise regression models predicting PARCA book.

	PARCA Book^c^											
	Model 4				Model 5				Model 6			
	*B*	*SE* B	β	99.6% CI	*B*	*SE* B	β	99.6% CI	*B*	*SE* B	β	99.6% CI
Gender	-.14	.11	-.09	[-.46, .18]	-.14	.11	-.09	[-.46, .18]	-.14	.11	-.09	[-.45, .18]
SES	.04	.10	.03	[-.25, .33]	.04	.10	.03	[-.25, .33]	.04	.10	.03	[-.25, .32]
Children's age	**.52**	**.06**	**.64**	**[.36, .69]**	**.51**	**.06**	**.63**	**[.34, .69]**	**.52**	**.06**	**.64**	**[.36, .69]**
AWC^a^	**.09**	**.02**	**.29**	**[.03, .16]**	**.09**	**.02**	**.29**	**[.03, .16]**	**.09**	**.02**	**.29**	**[.03, .16]**
ALD^b^	.10	.06	.13	[-.07, .27]	.10	.06	.13	[-.07, .27]	.10	.06	.13	[-.07, .27]
AVS^b^	.07	.06	.09	[-.11, .25]	.07	.06	.09	[-.11, .25]	.07	.06	.09	[-.11, .25]
Age*AWC^a^	-.01	.02	-.02	[-.07, .06]								
Age*ALD^b^					-.02	.54	-.03	[-.18, .14]				
Age*AVS^b^									-.04	.05	-.05	[-.17, .10]
*R*^2^	.54			.54			.54					
*F*	18.35			18.39			18.53					
*p*	< .001			< .001			< .001					

*Note.* PARCA = Parent Report of Children's Abilities; SES = socioeconomic status; AWC = adults’ word counts; ALD = adults’ lexical diversity; AVS = adults’ vocabulary sophistication; CI = confidence interval. Variables corrected for ^a^ recording duration

b number of available recordings. ^c^*N* = 104. Predictors significant at *p* < .004 are shown in bold. Each models’ *p* value is adjusted for 12 comparisons.

**Table 9:** Stepwise regression models predicting parent report.

	Parent Report											
	Model 1				Model 2				Model 3			
	*B*	*SE* B	β	99.6% CI	*B*	*SE* B	β	99.6% CI	*B*	*SE* B	β	99.6% CI
Gender	-1.72	.62	-.26	[-3.55, .11]	-1.14	.53	-.17	[-2.72, .43]	-1.21	.55	-.18	[-2.82, .40]
SES	.47	.55	.08	[-1.16, 2.10]	.65	.47	.11	[-.74, 2.03]	.52	.48	.09	[-.89, 1.93]
Children's age					**1.73**	**.27**	**.52**	**[.94, 2.52]**	**1.65**	**.27**	**.50**	**[.84, 2.50]**
AWC^a^									.06	.11	.04	[-.27, .39]
ALD^b^									-.28	.29	-.09	[-1.14, .58]
AVS^b^									.46	.30	.14	[-.43, 1.34]
Age*AWC^a^												
Age*ALD^b^												
Age*AVS^b^												
*R*^2^	.06			.32			.32					
*F*	4.15			17.65			9.36					
*p*	.22			< .001			< .001					

*Note.* SES = socioeconomic status; AWC = adults’ word counts; ALD = adults’ lexical diversity; AVS = adults’ vocabulary sophistication; CI = confidence interval. Variables corrected for ^a^ recording duration

b number of available recordings. Predictors significant at *p* < .004 are shown in bold. Each models’ *p* value is adjusted for 12 comparisons.

**Table 10:** Stepwise regression models predicting parent report.

					Parent Report							
	Model 4				Model 5				Model 6			
	*B*	*SE* B	β	99.6% CI	*B*	*SE* B	β	99.6% CI	*B*	*SE* B	β	99.6% CI
Gender	-1.23	.54	-.19	[-2.84, .37]	-1.25	.55	-.19	[-2.86, .37]	-1.26	.54	-.19	[-2.85, .34]
SES	.44	.48	.07	[-.98, 1.86]	.53	.48	.09	[-.88, 1.94]	.52	.47	.09	[-.87, 1.91]
Children's age	**1.61**	**.27**	**.49**	**[.80, 2.42]**	**1.70**	**.28**	**.51**	**[.87, 2.53]**	**1.61**	**.27**	**.49**	**[.82, 2.42]**
AWC^a^	.05	.11	.04	[-.28, .38]	.06	.11	.05	[-.27, .40]	.07	.11	.05	[-.26, .40]
ALD^b^	-.28	.29	-.08	[-1.13, .58]	-.25	.29	-.08	[-1.12, .61]	-.26	.29	-.08	[-1.11, .60]
AVS^b^	.48	.30	.15	[-.40, 1.37]	.45	.30	.14	[-.44, 1.34]	.43	.30	.13	[-.45, 1.31]
Age*AWC^a^	.13	.10	.10	[-.17, .44]								
Age*ALD^b^					.22	.27	.07	[-.59, 1.03]				
Age*AVS^b^									.38	.22	.14	[-.25, 1.02]
*R*^2^	.33			.32			.34					
*F*	8.31			8.09			8.65					
*p*	< .001			< .001			< .001					

*Note.* SES = socioeconomic status; AWC = adults’ word counts; ALD = adults’ lexical diversity; AVS = adults’ vocabulary sophistication; CI = confidence interval. Variables corrected for ^a^ recording duration

b number of available recordings. Predictors significant at *p* < .004 are shown in bold. Each models’ *p* value is adjusted for 12 comparisons.

## Discussion

Using data from naturalistic home observations, we tested if children's age moderated associations between characteristics of adult speech and children's language and cognitive outcomes. In line with previous studies (e.g., Caskey et al., 2014; Hart & Risley, 1995; Weizman & Snow, 2001), we found positive associations between some characteristics of adult speech and children's language and cognitive outcomes (i.e., main effects). However, we found no support for the hypothesis that children's age moderated these associations. This finding fails to substantiate earlier findings that the quantity of adult speech influences children's early language development but later, the diversity and sophistication of the language input become more important (Rowe, 2012).

### Associations between characteristics of adult speech and children's outcomes

We found that the quantity of adult speech accounted for 8% of the variance in children's PARCA booklet scores, suggesting that children who were exposed to a greater amount of language had better cognitive ability. This finding aligns with earlier studies that reported a positive association between the quantity of language input and children's cognitive ability at 3 years old (Hart & Risley, 1995). In a sample of preterm infants, the quantity of adult speech that they experienced at age 9 months accounted for 26% of the variance in their cognitive ability scores 10 months later (Caskey et al., 2014). However, another study with 30 children aged 12 to 20 months did not find a similar association (Greenwood et al., 2011).

It is plausible that the exposure to a large number of words provides more learning opportunities for children that help enhance their general cognitive development. However, the quantity of adult speech was not associated with children's cognitive ability when it was rated by parents. It is possible that the two components of the PARCA are capturing slightly different aspects of cognitive ability. This notion is supported by Saudino et al. (1998) who report that each component of the PARCA contributes unique prediction to the Mental Development Index of the Bayley Scales of Infant Development-II (Bayley, 1993). Perhaps the quantity of language input supports one area of cognitive development more strongly than another. To our knowledge, no previous study has assessed the association between adult word counts and the two components of the PARCA.

The lexical diversity of adult speech was not associated with children's cognitive or language outcomes. Because we applied a conservative adjustment for multiple comparisons to our regression models, the association between adults’ and children's lexical diversity was non-significant. Without this adjustment, adults’ lexical diversity was a significant predictor at *p* < .01 of children's lexical diversity, explaining 6% of the variance. This effect size is comparable Rowe's (2012) study, which did not adjust for multiple comparisons and reported that adults’ lexical diversity explained 9% of variance in children's lexical diversity.

We found that adults’ vocabulary sophistication accounted for 28% of the variance in children's concurrent vocabulary sophistication. By comparison, Rowe (2012) reported that the amount of adults’ vocabulary sophistication that children heard at 30 months explained 6% of the variance in their own vocabulary sophistication one year later. It is possible that this relatively strong association in our study was in part due to discourse effects, whereby the topic of conversation influences speakers’ vocabulary sophistication, as both the adults’ and children's vocabulary sophistication measures were derived from the same audio excerpts. Bidirectional effects may also underlie the associations observed here, for example, children with more sophisticated vocabulary may elicit more sophisticated speech from adults, and vice versa. However, it is also plausible that children exposed to a large variety of sophisticated words have more opportunity to learn these words, which they can then embed into their own productive vocabulary (Weizman & Snow, 2001).

Adults’ lexical diversity moderately correlated with their vocabulary sophistication, which suggests that these are distinct, yet inter-related language markers. By comparison, the quantity of adult speech was not related to adults’ lexical diversity or to their vocabulary sophistication, which highlights that these measures reflect distinct aspects of speech. This may be because the quantity of adult speech represents three days of interactions between a larger number of speakers than the diversity and sophistication measures. In other words, the quantity of adult speech reflects more broadly children's daily language environments, rather than the times of heightened language interactions.

We found no significant association between the diversity and sophistication of children's spoken language. This finding corroborates the notion that these two measures are tapping into distinct aspects of vocabulary richness (Malvern et al., 2004), at least in young children.

### Children's age as a moderator

Previous studies suggested that children's age moderates the association between characteristics of adult speech (i.e., the lexical diversity and vocabulary sophistication) and children's own language (Jones & Rowland, 2017; Pan et al., 2005; Rowe, 2012). Once children have a basic vocabulary, the exposure to a larger variety of different and rare words increases the opportunity for word-learning. While we found no evidence for a moderation effect, children's age added to our models’ prediction by 14%, 40% and 27% for children's lexical diversity, their PARCA booklet performance and their parents’ ratings of cognitive development, respectively. By comparison, children's age did not add to the model for child vocabulary sophistication.

Our study differed from previous studies on three key attributes: first, we assessed all speech heard by the child, whereas the previous studies measured maternal speech directed to the child (Jones, & Rowland, 2017; Pan et al., 2005; Rowe, 2012). Because our study used unobtrusive audio-recordings with excerpts selected, unknown to the families, for the highest conversational interactions between adults and children, it is more representative of natural language environments than solely focusing on maternal-child interactions. Second, we computed D-scores as our measure of children's lexical diversity akin to Jones and Rowland (2017), yet Pan et al. (2005) used word types, which are influenced by the number of words in the sample of speech and thus it is not an independent measure. Opting for another alternative assessment, Rowe (2012) assessed children's receptive vocabulary via a standardized test, which measures words that children know or understand. Receptive vocabulary is contrasted with expressive vocabulary, which refers to words that children can actually say. This differentiation reflects that in language development, children may understand a word but cannot (yet) vocalize it. Naturalistic observations of expressive vocabulary, like in our study, are reliant on the child spontaneously producing the words, which may not happen during a recording session even though the child can vocalize the word.

Third, we assessed concurrent interactions between adults and children and thus, the associations between adult and child measures may be bidirectional. By comparison, the earlier studies were longitudinal, with the times between assessments varying from 5 to 22 months. Because our study was cross-sectional the child age variable also encompassed inter-individual variation: children differed not only on age but also on other unexamined factors. We attempted to address this issue by controlling for SES and gender in our analysis but nevertheless, we were unable to control for all potential confounds. For example, children of the same age have different levels of language ability because children acquire language at different rates (Kidd et al., 2018). Therefore, it may be that the quantity of adult speech is more influential than other markers of language input during early language learning, however some children in our sample may have progressed beyond this and thus children's language ability rather than child age may be the moderator between language input and child outcomes (d'Apice, 2019).

Only Pan et al. (2005) formally tested the interaction between children's age and characteristics of adult speech on children's language. Both Jones and Rowland (2017) and Rowe (2012) compared associations between earlier input and later child language, whilst controlling for children's earlier vocabulary in their analysis. Because we were unable to adjust for children's previous language ability, we would expect that the associations between language input and children's language outcomes in our study are higher than those previously reported, as the variance explained by earlier ability would be subsumed within our child language outcome variables.

Although we attempted to address some of the limitations of previous research in this area, we did not observe an interaction effect between children's age and characteristics of adult speech in the prediction of children's cognitive and language skills. This finding has several theoretical implications, first, the interaction effect may not be immediately observable, and second, children's development may occur gradually rather than in stages.

First, children's age may indeed moderate the association between different markers of adult language input and children's outcomes however a time lag may exist before the moderation is detectable. This notion aligns with previous research that reported no concurrent association between mothers’ and children's lexical diversity at child age 28 months, however after five months, maternal lexical diversity explained 13% of the variance in children's growth in lexical diversity (Jones and Rowland, 2017). Similarly, Rowe (2012) reported that the diversity and sophistication of language input at child age 30 months was associated with children's growth in receptive vocabulary one year later.

A second possible explanation for the lack of an interaction effect is that child development is a continuous process rather than occurring at distinct critical periods (i.e., ages). Therefore, the influence of different markers of language input is important and consistent across early life. For example, lexically diverse and sophisticated language input comprises an abundance of phonemes from which children can build their vocabulary throughout the early years.

We suggest that future work in this area would benefit from careful methodological considerations. Digital technologies enable collecting naturalistic home observations over long durations (i.e., days) that are free from observer biases, resulting in rich 'big' data. Moreover, digital technologies can better afford collecting such 'big' data from large, well-powered samples than is possible with trained researchers that conduct multiple home visits over time.

### Limitations

Our study has many strengths but also several limitations. First, because our study design was cross-sectional, we could not control for children's prior cognitive or linguistic ability, examine individual growth trajectories, or model potential bidirectional effects. Our study design also implies that the observed age-related differences may be confounded by children's differences in cognitive and language abilities. Second, we relied on 30 min of observations for our assessment of lexical diversity and vocabulary sophistication, which may be too short to get an accurate representation of children's language abilities, although this observation period is comparable to previous studies (Pan et al., 2005). In addition, we did not distinguish overheard from child-directed speech, the latter of which is known to benefit more strongly child language development. Third, we derived adults’ and children's lexical diversity and vocabulary sophistication measures from the same transcripts and thus, they may be influenced by discourse effects that make their observations dependent on one another. Fourth, we applied a very stringent adjustment for multiple comparisons to our regression models which is likely to increase the risk of type II errors. However, even without the Bonferroni correction, no significant interactions were detected, suggesting that the correction itself did not lead to type II errors. Fifth, Tabachnick and Fidell (2014) suggest using a sample size of 104 plus the number of predictors, to test the influence of the predictors on a dependent variable in a regression model when there is a medium effect size. For our full regression models, our sample size of 107 falls slightly short of their recommendation (i.e., 104 + 7 = 111). Nevertheless, our sample size is comparable to other research in this field.

## Conclusions

Using unobtrusive audio recordings, we showed that characteristics of adult speech are associated with children's language and cognitive skills. Our findings suggest that these associations do not vary meaningfully across children's age. We encourage future studies in this area to adopt longitudinal designs and collect rich naturalistic observations to explore the importance of adult speech for children's cognitive and language outcomes throughout their developmental stages.

## Ethics

Informed consent was obtained. The research was conducted in accordance with APA ethical standards in the treatment of the study sample.

## CRediT authorship contribution statement

**Katrina d'Apice:** Conceptualization, Methodology, Software, Formal analysis, Investigation, Data curation, Writing – original draft, Writing – review & editing, Visualization, Project administration. **Sophie von Stumm:** Conceptualization, Methodology, Software, Resources, Supervision, Funding acquisition, Writing – review & editing, Project administration.

## Declarations of Competing Interest

None.

## Data Availability

The data that has been used is confidential.
